# Ultrasound-Guided Saphenous Nerve Block in Rabbits (*Oryctolagus cuniculus*): A Cadaveric Study Comparing Two Injectate Volumes

**DOI:** 10.3390/ani12050624

**Published:** 2022-03-01

**Authors:** Ricardo Felisberto, Derek Flaherty, Hamaseh Tayari

**Affiliations:** Southern Counties Veterinary Specialists (SCVS), Forest Corner Farm, Hangersley, Hampshire, Ringwood BH24 3JW, UK; ricardo.felisberto@scvetspecialists.co.uk (R.F.); derek.flaherty@scvetspecialists.co.uk (D.F.)

**Keywords:** loco-regional anesthesia, analgesia, rabbit, ultrasound-guided, saphenous nerve block, interfascial block

## Abstract

**Simple Summary:**

General anesthesia in rabbits is associated with significantly higher mortality than in dogs and cats. In addition, as prey animals, rabbits tend to mask signs of pain, making early detection particularly difficult. Loco-regional anesthesia represents a fundamental component of a multimodal approach to pain management and is an effective strategy to reduce the need for systemic anesthetic and analgesic drugs, thereby limiting their associated side effects. The saphenous nerve is the largest sensory branch of the femoral nerve and provides sensory supply to areas of the hind-limb. Saphenous nerve blockade allows for analgesia of the front and inner parts of the pelvic limb without affecting femoral nerve motor function, thus allowing optimal pain relief but permitting continued mobilization, which may be of particular importance in rabbits as it allows preservation of normal physiological prey behavior (e.g., hiding, kicking). This cadaveric study describes an ultrasound-guided saphenous nerve block technique in rabbits and compares the length of the nerves stained following injection of two different dye volumes. The results show that both volumes consistently stained the saphenous but not the femoral nerve. This technique has the potential to provide hind-limb analgesia while preserving femoral motor function in rabbits.

**Abstract:**

Ultrasound-guided (US-guided) loco-regional anesthesia techniques allow direct visualization and blockade of sensory nerves. The saphenous nerve (SN), a terminal branch of the femoral nerve (FN), is strictly a sensory nerve for which electrical locator devices are ineffective for localization as no effector muscle contractions can be evoked. US-guided SN block in species other than rabbits produces hind-limb analgesia without affecting FN motor function. The aims of this study were to develop a US-guided SN block technique in rabbits and to compare the spread obtained using two different dye volumes. Twelve hind-limbs from six cadavers (1.6 ± 0.1 kg) were included; after randomization, the SN block was performed on the right or left hind-limb, injecting 0.05 mL kg^−1^ or 0.1 mL kg^−1^ of tissue dye in lidocaine (1:50 *v*:*v*). Subsequent dissections allowed nerve staining measurements. All SNs were identified, and 17.8 ± 4.6% and 31.0 ± 8.9% of the SN length were stained using low-volume and high-volume of the dye, respectively. Regardless of the volume used, the SN was consistently stained while the motor branch of the FN was not. This US-guided technique may provide hind-limb analgesia without affecting FN motor function in rabbits undergoing mid-distal hind-limb surgeries.

## 1. Introduction

Unlike dogs and cats, rabbits have been domesticated for only a relatively short time; however, as their popularity as companion animals increases, there is greater demand for the provision of anesthesia in this species [1]. Unfortunately, possibly related to their peculiar anatomical, physiological, and behavioral features, there is a higher risk of peri-anesthetic death in rabbits (between 1.39% [2] to 4.8% [1]), compared to cats (0.24%) and dogs (0.17%) [2]. In most cases, the primary etiology of peri-anesthetic death is unknown but is often ascribed to either cardiovascular or respiratory causes [1,2]. Additionally, a high incidence of non-fatal gastrointestinal complications is also common in rabbits (38%) [1]. No specific risk factor has been associated with the higher peri-operative mortality rate recorded in rabbits, and this is most likely multifactorial (e.g., dietary changes, pain, disease, and medication side effects) [3].

Prey animals such as rabbits are reluctant to show signs of discomfort, making them particularly challenging for early detection of pain as well as the evaluation of the efficacy of an analgesic treatment if this is based on behavioral changes [1,4,5,6,7]. Therefore, adequate peri-operative analgesia plays a vital role in the outcome [4,5,6]. While no single technique or drug regimen has been shown to eliminate peri-anesthetic morbidity and mortality in any species, a pre-emptive analgesia strategy and a multimodal approach to pain management should ensure optimal peri-operative analgesia in rabbits, reducing the risk of under-detection of pain and the occurrence of possible related complications in the peri-anesthetic period, such as ileus [1,4,5,8].

Loco-regional anesthesia techniques, such as peripheral nerve blocks (PNB) and interfascial blocks, are fundamental components of a multimodal approach to pain management and are effective in reducing the need for systemically-administered analgesic and anesthetic drugs, potentially limiting the latters’ side effects [9,10]. However, in addition to preventing the transmission of nociceptive signals to the cortex, many loco-regional techniques will also result in a degree of motor block that may affect the rabbit’s normal behavior pattern. Temporary absence of physiological prey behaviors may increase hospitalization-associated stress, reduce food intake, and potentially increase the risk of stress-related complications, such as ileus [1]. Furthermore, prolonged immobility during the post-operative period secondary to impaired motor function may limit efficient ventilation, as reported in humans [11]; this may be particularly significant in rabbits, where there is a high incidence of pre-existing pulmonary disease, even in apparently healthy individuals [1].

Hind-limb innervation in dogs derives from the sciatic nerve and femoral nerve (FN); the latter runs inside the iliopsoas muscle and receives contributions from the fourth, fifth, and sixth lumbar spinal cord segments (L4–L6) [12]. Before leaving the iliopsoas muscle, the FN gives origin to the saphenous nerve (SN) and is located cranially to the femoral artery (FA) in a different interfascial plane [9]. The SN runs distally, cranial to FA, and within the same fascia (medial femoral fascia), forming a so-called ‘neurovascular bundle’ [9,13,14]. The SN supplies branches to the stifle joint and ends in the skin over the first digit (when present) [14,15]. Overall, in dogs, the SN is responsible for the sensory innervation of the medial aspect of the distal thigh, stifle joint, tibia, tarsus, and metatarsus, and the cranial aspect of the stifle [13].

Similarly, hind-limb sensory and motor innervation in rabbits is provided by the FN and sciatic nerve, which originate from the lumbosacral plexus (L4–L7), and the first and second sacral cord segments (S1–S2) [16,17,18].

In dogs, the peripheral block of the SN has been shown to provide desensitization of the medial and cranial aspects of the distal limb (including the stifle) without affecting the motor function of the quadriceps femoris muscle [9,19]. Due to the neuro-anatomical similarities between dogs and rabbits [16,17,19,20], it is likely that this also applies to rabbits. Two previous studies reported successful blockade of FN (and sciatic nerve) using electrical stimulation in rabbits undergoing pelvic limb orthopedic procedures [17,18]. However, by blocking the FN (instead of SN), the motor function of the quadriceps, gastrocnemius, and cranial tibial muscles [16] will potentially be impaired during the post-operative period, affecting voluntary movements and possibly impacting negatively on the animal’s welfare.

Electrical locator devices are ineffective in localizing sensory nerves since motor fibers are lacking in these types of nerves and muscle contractions cannot be evoked [9,19]. To perform a SN block, as well as other sensory nerve blocks, ultrasound-guided (US-guided) techniques should be applied, as direct visualization of the targeted nerve and landmarks is possible. Besides these advantages, the US-guided techniques also allow real-time visualization of the local anesthetic spread around the nerves while reducing the risk of damage to other vital structures (e.g., arteries or veins) [9,19].

In dogs, the US-guided SN block technique has been designed as an interfascial block injection, as the local anesthetic is injected into the medial femoral fascia that contains the SN and both the FA and FN [9,19].

Recently, a case report describing the use of a combined US-guided and nerve stimulation-guided saphenous and sciatic nerves blocks in a pet rabbit undergoing calcaneal fracture repair demonstrated the effectiveness in peri-operative pain management with no impairment of motor function in the affected limb [20].

To the authors’ knowledge, there are no previous studies that have investigated the sono-anatomy of the SN and its surrounded structures in rabbits and designed a US-guided SN block technique specifically for this species. Additionally, no previous research in companion animals has investigated the resulting length of SN stained once the US-guided SN block technique is performed and if this would affect the femoral motor branch (FMB). The aims of the present study were (1) to describe the gross anatomy of the SN and surrounded structures in rabbits; (2) to describe the sono-anatomy of the SN in relation to the surrounding structures and to design a US-guided SN block technique specifically for rabbits; and (3) to compare the length of the nerves (saphenous and femoral motor branch) stained after injecting two different dye volumes (low volume, 0.05 mL/kg; high volume, 0.1 mL/kg). Our hypotheses were that the SN could be easily identified using the US-guided technique and that the spread obtained with both injected dye solution volumes would effectively stain the SN while not diffusing to the FMB, which may potentially preserve the motor function in vivo.

## 2. Materials and Methods

This study included a total of six cadavers of adult Californian rabbits *(Oryctolagus cuniculus)*. No animals were euthanized for the purpose of this study, and no ethical committee approval was required as this was purely a cadaveric study. The rabbits were obtained frozen from a certified local butchery and were slowly thawed at room temperature over a period of 24 h. All cadavers were presented skinned, partially eviscerated, and exsanguinated. Cadavers that could still be frozen (based on palpation) or with damaged vertebrae, pelvic bones, nerves, muscles, or other inguinal structures were excluded.

### 2.1. Gross Anatomical Investigation

**Figure 1:**
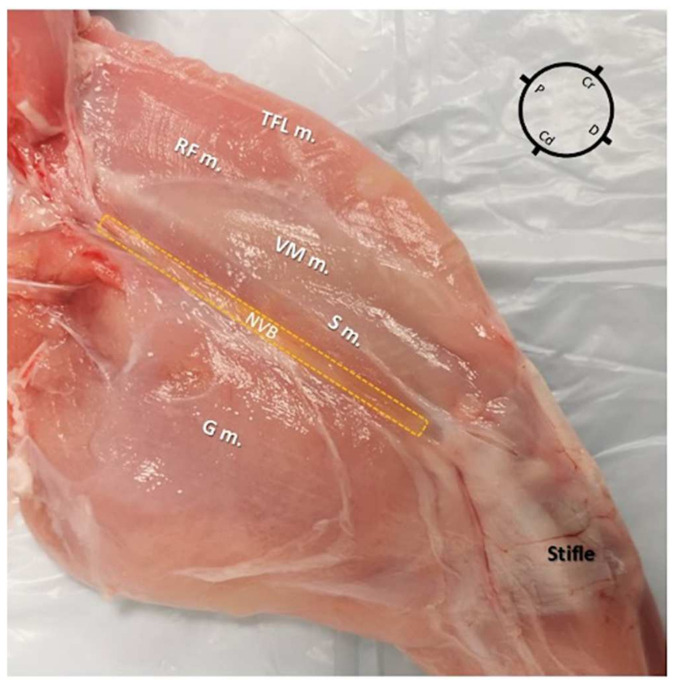
Skinned rabbit hind-limb positioned in dorsal recumbency with the hind-limb extended in a natural position for gross anatomical dissection (medial aspect of the left hind-limb). At the anterior aspect of the pelvic limb, the vastus medialis (VM m.), sartorius (S m.), rectus femoris (RF m.), and tensor fascia latae (TFL m.) muscles were identified. At the posterior aspect of the hind-limb the gracilis muscle (G m.) was identified. In the medial-center aspect of the thigh, a depression containing the neurovascular bundle (NVB) (yellow dotted lines area) was identified. P: Proximal; D: Distal; Cr: Cranial; Cd: Caudal.

The first cadaver (2 hind-limbs) was used only for gross anatomical dissections in order to evaluate the SN anatomy and its relationship with the surrounding muscles and vessels in the rabbit. Both hind-limbs were dissected with the rabbit cadaver placed in dorsal recumbency and the hind-limb extended in a natural position but externally rotated around the longitudinal plane. For each hind-limb, following the identification of the superficial muscles (Figure 1), dissection was performed by removing the vastus medialis, sartorius, gracilis, and adductor muscles before visualizing the medial femoral fascia containing the neurovascular bundle. After identification of the iliopsoas muscle, the origin of the FN was identified and its path was followed along the medial aspect of the hind-limb. The FN and its branches, the SN, and motor branch (FMB), were also identified.

### 2.2. Sono-Anatomy Study and US-Guided Saphenous Nerve Block Design

**Figure 2:**
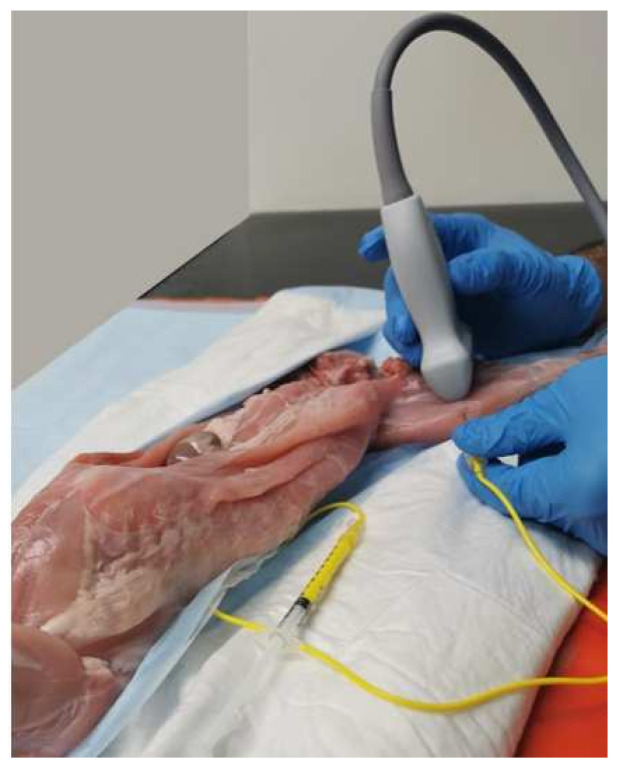
Representation of the position of the ultrasound transducer over the medial aspect of the middle of the thigh, transversally to the long axis of the pelvic limb. The probe is positioned with the marker caudally to visualize the saphenous nerve.

The other five cadavers (10 hind-limbs) were used for sono-anatomy investigation of the SN and surrounding structures in order to design the US-guided SN block. For all 10 legs, the medial aspect of the hind-limb was first ultrasound screened using a veterinary dedicated ultrasound system (Fujifilm Sonosite lnc., S II Veterinary Ultrasound System, Bothell, WA, USA). Ultrasound gel (Aquasonic 100, Parker, Fairield, NJ, USA) was used to establish contact between the ultrasound probe (L25x, 13-6 MHz Linear Transducer, Bothell, WA, USA) and the muscle layers. The ultrasound probe was placed over the proximal inguinal area, transversally to the long axis of the pelvic limb, where the FN was visualized before branching into the SN and FMB. The US probe was then slid distally to the level of the middle thigh, where the acoustic window displayed the femur, the vastus medialis, adductor, pectineus, and sartorius muscles, and the neurovascular bundle (Figure 2). At this level, the terminal portion of the pectineus muscle was visualized. Based on our gross anatomical study, all these aforementioned structures were considered as landmarks to design the US-guided SN block technique. In order to consistently perform the US-guided SN block in all cadavers, the terminal portion of the pectineus muscle was then chosen as the main landmark. To better visualize the targeted acoustic window, adjustments were performed with minor ultrasound probe movements (minor rotation and minor sliding movements) until the neurovascular bundle was visualized at the center of the US window, caudally to the vastus medialis muscle and femur, cranially to the adductor and pectineus muscle, and below the sartorius muscle.

After each sono-anatomy study, the hind-limb was assigned using an online randomizer generator (www.random.org, accessed on 5 February 2022) to receive either a low (0.05 mL/kg) or high (0.1 mL/kg) volume of the injectate, which comprised a mixture of a permanent tissue dye solution (Tissue marking dye yellow, Mopec, Madison Heights, MI, USA) with lidocaine 2% (Lidocaine, Hameln Pharmaceuticals, Gloucester, UK) mixed in a 1:50 *v*:*v* ratio [21].

For the US-guided SN block technique, a 21-gauge, 50 mm echogenic insulated needle (Echoplex, Vygon, Swindon, UK) with an attached extension line primed with the injectate solution was inserted through the tensor fascia latae muscle in a cranio-caudal orientation towards the neurovascular bundle. The along visual axis technique (the long axis of ultrasound probe was along the operator’s visual axis and ultrasonic beam and vertical to the surface) and in-plane needling approach were used to perform the US-guided SN block with lidocaine-dye mixture injections in all cadavers [22]. The position of the tip of the needle was deemed satisfactory when it pierced the medial femoral fascia containing the SN and the FA and Femoral vein (FV). This technique was consistently applied to all cadavers for all hind-limbs by the same operator (R.F.).

### 2.3. Anatomical Dissection following US-Guided Saphenous Nerve Block

The rabbit cadavers were kept in dorsal recumbency and a sagittal incision was made from the inguinal crease to the medial aspect of the stifle along the center of the thigh. Careful blunt dissection was performed to remove the sartorius, gracilis, vastus medialis, and adductor magnus muscles. This allowed the exposure of the pectineus and iliopsoas muscles, the FA and FV, and the FN and its branches (motor and SN branches). Following this, blunt dissection of the FA, FV, and of the SN allowed their separation from the surrounding structures, from the inguinal crease to the proximal level of the stifle. The medial femoral fascia containing these structures was preserved to allow the evaluation of the spread limits of the dye solution and whether the SN and the femoral motor branch (FMB) were stained in the hind-limbs injected with either the low dye solution volume (0.05 mL/kg) or with the high volume (0.1 mL/kg).

The SNs were considered to have been successfully stained when the dye solution was detected within the medial femoral fascia and around the entire circumference of the SN for a length of ≥1 cm [23], but not outside the fascia. The total length of the SN was considered from the bifurcation of the FN to the proximal level of the stifle joint. The nerves’ staining length was measured in all cadavers with a standard ruler.

### 2.4. Statistical Analysis

Statistical analysis was performed using Microsoft Excel (v16.0, Microsoft Corporation, Santa Rosa, CA, USA). The parametric data were tested for normal distribution by the Shapiro-Wilk test and are presented as mean ± standard deviation (SD). The Fisher’s exact test was performed to determine statistical significance using two categories, category-1 SN stained for ≥1 but ≤2 cm, category-2 SN stained for >2 cm [12,23]. A *p*-value less than 0.05 was considered statistically significant.

## 3. Results

All cadavers were in satisfactory condition after thawing, and no significant damage was observed; therefore, all cadavers were included in the study. Due to the cadaveric preparation, it was not possible to determine the sex or age of the rabbits; however, the mean carcase weight was 1.6 ± 0.1 kg.

### 3.1. Gross Anatomical Investigation

**Figure 3:**
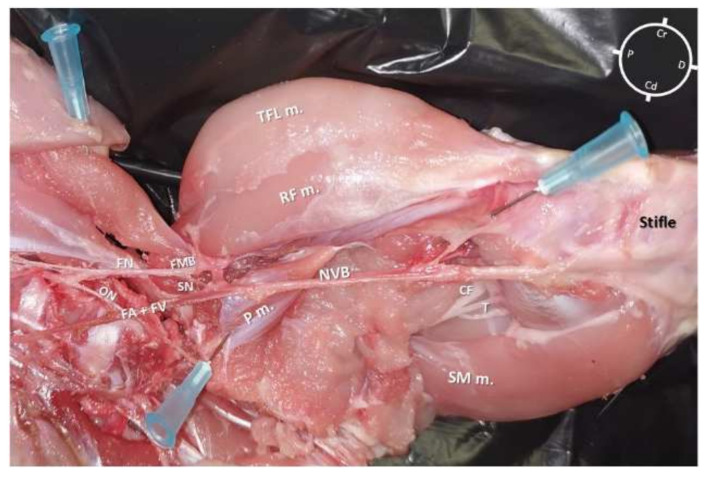
Skinned rabbit hind-limb positioned in dorsal recumbency with the hind-limb extended in a natural position after blunt dissection of the medial aspect of the left thigh and removal of the vastus medialis, sartorius, gracilis, and adductor muscles. The femoral nerve is observed leaving the psoas compartment and, at the level of the inguinal crease, giving origin to a motor branch that runs into the muscles located in the anterior aspect of the thigh and to the saphenous nerve, which joins into the same fascia of the femoral artery and vein forming the neurovascular bundle (NVB). This bundle runs medial and caudal to the femur, caudal to the vastus medialis, rectus femoris (RF m.), and tensor fascia latae (TFL m.) muscles, cranial to the adductor, semimembranosus (SM m.), and gracilis muscles and below the sartorius muscle. P: Proximal; D: Distal; Cr: Cranial; Cd: Caudal; FN: Femoral nerve; ON: Obturator nerve; SN: Saphenous nerve; FA + FV: Femoral artery + Femoral vein; FMB: Femoral motor branch; P m.: Pectineus muscle; CF: Common fibular nerve; T: Tibial nerve.

In both hind-limbs, it was possible to observe the FN giving origin to the SN and to the FMB after leaving the psoas compartment at the proximal inguinal area. At this level, the FMB ran to the anterior aspect of the thigh, where it gave origin to other branches. The SN was identified within the medial femoral fascia as part of the neurovascular bundle, cranially to the FA and FV. The SN was localized below the sartorius muscle caudally to the rectus femoris and vastus medialis muscles and cranial to the pectineus and adductor muscles. In the middle compartment of the thigh, the pectineus muscle was identified as a triangle-shaped muscle located below the neuromuscular bundle. The distal insertion of the pectineus muscle was located at the end of the first proximal third of the femur on its posteromedial surface. The femur was located cranially to the neurovascular bundle, adductor, semimembranosus, and pectineus muscle, caudally to the FMB, rectus femoris, tensor fascia latae muscles, and laterally to the vastus medialis muscle. Distally, at the proximal aspect of the stifle joint, the SN ran deeper to innervate the articular capsule (Figure 3).

### 3.2. Sono-Anatomy Study and US-Guided Saphenous Nerve Block

Despite all cadavers being considered correctly thawed for the anatomical study (based on palpation), in one of the hind-limbs, a mild degree of ice crystallization was identified in the middle thigh muscles during the sonography. However, as this finding did not interfere with the identification process of the acoustic target window and the neurovascular bundle, this hind-limb was also included.

In all hind-limbs, it was possible to identify the targeted acoustic windows at the level of the middle thigh using the landmarks as described in the anatomical study. The landmark structures (muscle, bone, and neurovascular bundle) were all visualized after mild probe movements within 1.5 cm depth for all cadavers.

**Figure 4:**
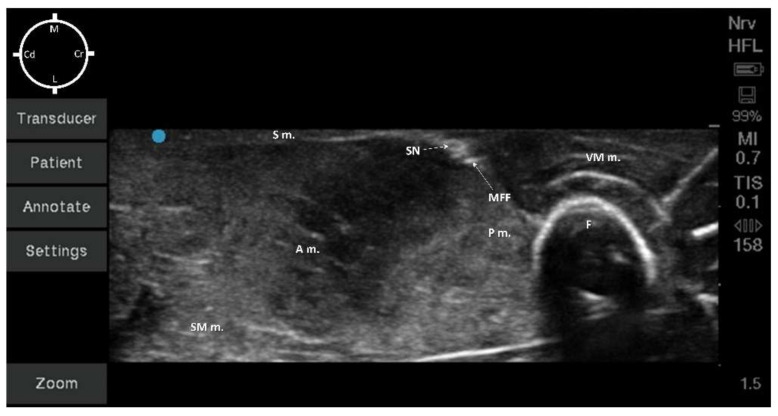
Sono-anatomy of the pelvic limb. The saphenous nerve (SN) can be visualized as a hyperechoic round structure, within the same fascial plane (medial femoral fascia) of other hyperechoic round structures (collapsed femoral artery and vein due to absence of blood flow). These structures were caudally to the vastus medialis muscle (VM m.), medially and caudally to the femur (F), cranially and medially to the adductor (A m.) and semimembranosus (SM m.) muscles, medially to the pectineus muscle (P m.) and immediately below the sartorius muscle (S m.). MFF: Medial Femoral Fascia; M: Medial; L: Lateral; Cr: Cranial; Cd: Caudal.

According to the anatomical findings, the US-guided SN block technique was designed, placing the ultrasound probe approximately at the central portion of the middle thigh transversally to the long axis of the hind-limb. At this level, the visualization of the neurovascular bundle was at the center of the acoustic window (Figure 4).

All the muscles (sartorius, adductor, semimembranosus, pectineus, and vastus medialis muscles) were displayed as structures with heterogeneous echogenicity. The femur was displayed as a hyperechoic structure with acoustic shadow. At the center of the targeted acoustic window, the terminal portion of the pectineus muscle was visualized as a triangular-shaped structure with heterogeneous echogenicity caudally to the femur. In addition, the neurovascular bundle was displayed as a piriform-shaped structure with a hyperechoic outer layer (medial femoral fascia) containing three hypoechoic round-shaped structures (SN, FA, and FV). It was not possible to confirm the position of the SN in relation to both the FA and FV due to the absence of blood flow (Figure 4). The neurovascular bundle was always located below the sartorius muscle and medially to the pectineus muscle within 0.5 cm depth for all cadavers. In addition, the vastus medialis muscle was located cranially, the adductor and the semimembranosus muscles were located caudally to the neurovascular bundle (Figure 4).

**Figure 5:**
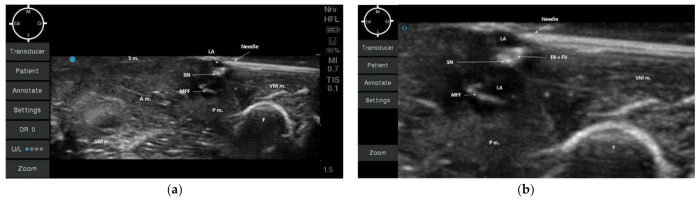
(**a**) Ultrasonographic image of the medial hind-limb in a rabbit cadaver at the level of medial femoral fascia (MFF). The saphenous nerve (SN) can be visualized as a hyperechoic round structure, next to other hyperechoic round structures (collapsed femoral artery and vein due to absence of blood flow), which are highlighted due to the presence of the anechoic dye solution (LA: permanent tissue dye solution and lidocaine 2% (1:50)); Once the tip of the needle pierced the medial femoral fascia, the dye solution was injected. (**b**) Highlighted ultrasound image of the injected solution within the fascia containing the saphenous nerve (using zoom option on the ultrasound device). S m.: Sartorius muscle; A m.: Adductor magnus muscle; SM m.: Semimembranosus muscle; P m.: Pectineus muscle; F: Femur; VM m.: Vastus medialis muscle; MFF: Medial Femoral Fascia; FA + FV: Femoral artery + Femoral vein; M: Medial; L: Lateral; Cr: Cranial; Cd: Caudal.

In all of the 10 hind-limbs, the US-guided SN block was feasible at the first attempt. The timing to successfully perform the block was around 5 min. During needling, the shaft of the needle was always visible, as was the tip when piercing the medial femoral fascia. The dye solution was injected in five hind-limbs with a low volume (0.05 mL/kg; resultant mean volume of 0.08 ± 0.006 mL) and in the other five with a high dose (0.1 mL/kg; resultant mean volume of 0.16 ± 0.009 mL). During injection, an anechoic area was formed within the medial femoral fascia, which improved the visualization of the SN for all cadavers (Figure 5a,b).

### 3.3. Anatomical Dissection following US-Guided Saphenous Nerve Block

**Figure 6:**
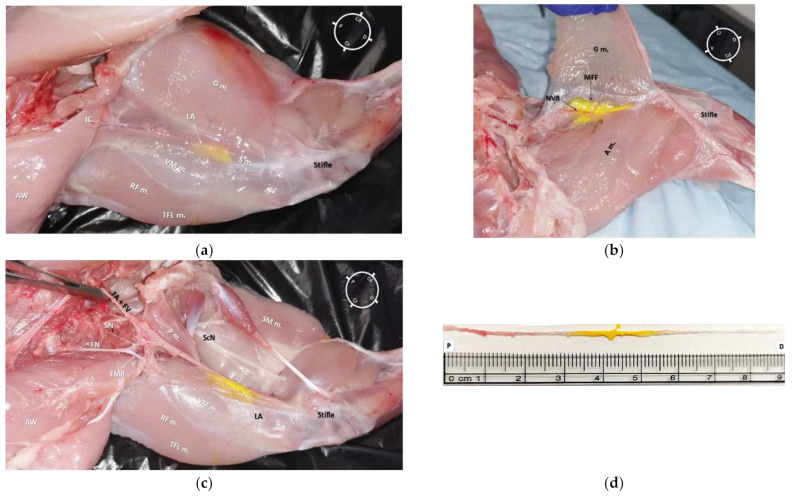
(**a**) Medial view of the right hind-limb of a rabbit cadaver in which a low volume dye solution (0.05 mL/kg, permanent yellow tissue dye solution and lidocaine 2% (1:50)) was injected. It is possible to observe the underlying tissues stained by dye solution in the central portion of the medial aspect of the thigh. (**b**) Medial view of the left hind-limb (different rabbit than (**a**)) of a rabbit cadaver after partial dissection of the gracilis muscle. It was possible to observe the distribution of dye solution inside the medial femoral fascia (MFF), which contains the saphenous nerve (SN) and both the femoral artery (FA) and vein (FV). (**c**) Anatomical dissection of the medial aspect of a left hind-limb. The gracilis, sartorius, and adductor magnus muscles were bluntly removed. It was possible to observe the dye solution within the medial femoral fascia staining the neurovascular bundle (SN, FA, and FV); the dye solution did not stain the femoral nerve (FN) and its motor nerve branches (FMB). It was possible to localize the pectineus muscle insertion onto the surface of the femur at the level of the first third of the thigh, which was caudal and lateral in relation to the SN. (**d**) The SN was completely removed from the cadaver (from where it branched off the FN to the proximal stifle, where it deepened to innervate the articular capsule) for the evaluation of its total length (9 cm) and extent of staining from the tissue dye solution (1.9 cm). S m.: Sartorius muscle; VM m.: vastus medialis muscle; G m.: gracilis muscle; TFL m.: tensor fascia latae muscle; RF m.: rectus femoris muscle IC: Inguinal crease; NVB: Neurovascular bundle; AW: Abdominal wall; LA: local anesthetic and tissue dye solution; P m.: pectineus muscle; ScN: sciatic nerve; SM m.: semimembranosus muscle; A m.: adductor magnus muscle; P: Proximal; D: Distal; Cr: Cranial; Cd: Caudal.

**Figure 7:**
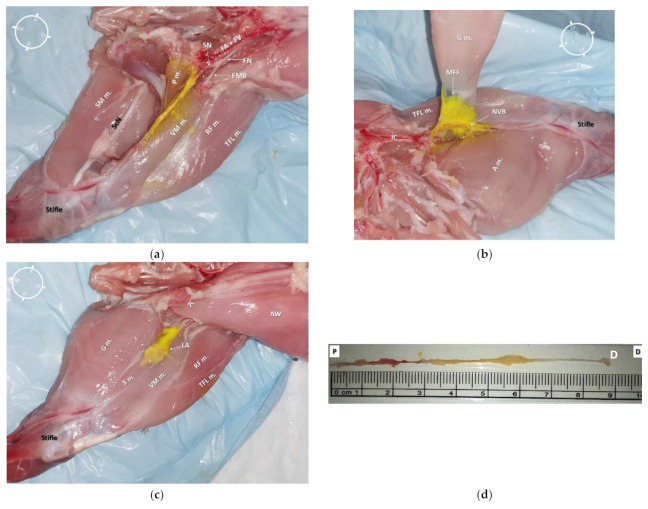
(**a**) Medial view of a left hind-limb of a rabbit cadaver in which a high volume of dye solution (0.1 mL/kg, of permanent yellow tissue dye solution and lidocaine 2% (1:50) was injected)). It was possible to observe the underlying tissues stained by the dye solution in the central portion of the medial aspect of the thigh. (**b**) Medial view of the same hind-limb after partial dissection of the gracilis muscle. It was possible to observe the distribution of the dye solution inside the medial femoral fascia (MFF), which contained the saphenous nerve (SN) and both the femoral artery (FA) and vein (FV). (**c**) Anatomical dissection of the medial aspect of the same hind-limb. The gracilis, sartorius, and adductor magnus muscles were bluntly removed. It was possible to observe the staining along the neurovascular structures (SN, FA, and FV) without staining both the femoral nerve (FN) and its motor nerve branches (the tissue dye stain on the vastus medialis and pectineus muscles resulted after opening of the medial femoral fascia during the dissection). It was possible to localize the pectineus muscle insertion onto the surface of the femur at the level of the first third of the thigh, which was caudal and lateral in relation to the SN. (**d**) The SN was completely removed from the cadaver (from where it branched off the FN to the proximal stifle, where it deepened to innervate the articular capsule) for the evaluation of its total length (8.7 cm) and extent of staining from the tissue dye solution (4.2 cm). S m.: sartorius muscle; VM m.: vastus medialis muscle; G m.: gracilis muscle; TFL m.: tensor fascia latae muscle; RF m.: rectus femoris muscle; P m.: pectineus muscle; IC: Inguinal crease; NVB: neurovascular bundle; AW: Abdominal wall; LA: local anesthetic and tissue dye solution; FMB: femoral motor branches; ScN: Sciatic nerve; SM m.: Semimembranosus muscle; A m.: adductor magnus muscle; P: Proximal; D: Distal; Cr: Cranial; Cd: Caudal.

In all cadavers, the dye solution was injected within the target area (medial femoral fascia) without involving the surrounding structures (Figure 6a and Figure 7a). It was possible to separate all the surrounding anatomical structures maintaining the neurovascular bundle integrity. The dye solution was visible within the medial femoral fascia containing the SN and both FA and FV in all of the 10 pelvic limbs (Figure 6b and Figure 7b). All SNs, regardless of the dye solution volume injected, were stained around their entire diameter and for more than 1 cm of length (Figure 6c,d and Figure 7c,d). In addition, none of the FNs nor FMB were stained with either high or low volumes of dye solution.

Individual total SN length, SN length of stain, and percentage of the total length of the nerve stained, reported as mean and standard deviation, are described in Table 1. The difference in the tissue dye solution spread along the SN was statistically significant between the two groups (*p* = 0.0476; Fisher’s Exact test).

## 4. Discussion

The present study is the first investigating the sono-anatomy of the middle thigh area in rabbits and the first to successfully design a US-guided SN block technique specifically for this species. In addition, it has also demonstrated that the length of the saphenous nerve stained was proportional to the dye solution volumes injected, but even the lower volume successfully stained the SN in a manner that should provide adequate blockade. The technique here described allowed the staining of the SN without involving the motor component of the FN (femoral motor branch). This US-guided SN block has the potential, therefore, to produce sensory blockade without affecting the motor function of the quadriceps femoris muscle in rabbits.

Based on this study, the anatomy and sono-anatomy of the medial aspect of the hind-limb and the relationship between the SN, femur, FA, FV, and muscles in rabbits are similar to that described in dogs [9,19]. However, in the rabbit, the pectineus muscle terminates on the proximal third of the femur, while in dogs, this occurs on the distal aspect [24]. In dogs, the main landmark for the US-guided SN block is the femoral artery [9,19]; however, due to the nature of this study in cadavers, other landmarks (muscles and femur) had to be used. The pectineus muscle was considered the main landmark in this study, as it allowed a consistent location to perform the US-guided SN block in all hind-limbs.

This technique could be considered as an interfascial plane block because the target point was the medial femoral fascia that contains the SN, and not directly the SN itself. Due to the small size of the SN in rabbits and to the proximity of vital structures (FA and FV), this block was designed as an interfascial block to reduce the risk of intravascular injection when performed in live animals, and also, therefore, a suitable option if the nerve (SN) is not clearly visualized or distinguished from the vascular structures. Furthermore, this may facilitate the contact of the nerve surface with the local anesthetic, as previously reported in cats [23].

The main landmark (pectineus muscle) and the target structures (neurovascular bundle inside the medial femoral fascia) were all within a maximal depth of 1 cm, which may have favored the visualization. Nonetheless, the small size of the SN in rabbits may make it challenging to perform the needling due to the proximity of the target structures to the ultrasound probe [25].

The volumes of the dye solution used in this study were decided according to previous similar studies performed in dogs targeting peripheral nerves [12,26], but lower than the volumes used to perform other interfascial plane nerve blocks in small animals (up to 0.6 mL/kg) [27,28,29]. In fact, in interfascial plane nerve blocks, the use of larger volumes of local anesthetic allows a more consistent spread around the targeted nerves, increasing the chances of successful nerve blockade [29]. In our study, although an adequate SN staining length was obtained in both groups, it is possible that ultrasound visualization and dye solution distribution within the medial femoral fascia may improve with the use of higher volumes. However, volumes of dye solution larger than those studied may spread to the FN and FMB; further research is required to investigate this. Nevertheless, the choice of the volume of injectate used should always account for the maximum dose of the local anesthetics allowed in the species considered [30].

In dogs, the use of 0.1 mL/kg of local anesthetic allowed complete sensory block of the saphenous nerve [19], while in our study, all SNs were adequately stained (complete staining around its diameter for more than 1 cm in length) [23], suggesting that this technique may result in an effective sensory block of the medial aspect of the mid-distal hind-limb even with a volume of 0.05 mL/kg. Future in vivo studies are needed to confirm these assumptions.

The present study has some limitations that need to be addressed. The echogenicity of fascial planes, muscles, and vascular structures may be altered during freezing and thawing and may not accurately reflect the sono-anatomy of the live rabbit. All cadavers were skinned, which may have improved the US visualization of the target structure and landmarks but may have made needling more challenging due to the proximity of the target structures to the probe [25].

The pressure of the injection was not measured during the US-guided SN block, and it is recognized that the spread of dye solutions within an interfascial plane may be unpredictable in cadavers due to differences in tissue integrity [31]. This may also influence the resistance to injection, which, if increased, could promote a wider spread of the dye solution when compared to live animals.

Further studies are needed to investigate whether the injection of a larger volume of dye solution using the described US-guided SN block technique would also reach the FN and FMB, producing motor block, or if performing the US-guided SN block more distally would lead to similar results to our study. In addition, clinical studies are required to evaluate the analgesic efficacy of this technique in rabbits.

## 5. Conclusions

In rabbits, the US-guided SN block herein described allowed a consistent adequate stain of the saphenous nerve in all cases without affecting the motor branch of the femoral nerve, even with the lower volume of dye solution (0.05 mL/kg). Furthermore, even with the higher volume (0.1 mL/kg) of dye solution, the femoral nerve and its motor branch were not stained in any case. This US-guided SN block technique (regardless of the volumes used) has the potential to produce a sensory blockade of the mid-distal hind-limb, including the medial and cranial aspect of the stifle, with no quadriceps muscle motor function impairment. This technique has the potential to preserve the rabbits’ normal behavior pattern in the post-operative period while reducing the risk of undetected pain; however, these conclusions need to be evaluated with in vivo studies.

## Figures and Tables

**Table 1:** Evaluation of the extent of nerve staining obtained by performing ultrasound-guided saphenous nerve (SN) blocks using a dye solution [permanent yellow tissue dye in lidocaine 2% (1:50)] in five rabbit cadavers (10 hind-limbs) with either low (0.05 mL/kg) or high (0.1 mL/kg) volume. Values (mean ± standard deviation) are reported for saphenous nerve total length, the length of the staining obtained, the total volume of dye solution injected for each hind-limb, and the percentage of the total length of saphenous nerves stained. SD: standard deviation; * The difference between the length of the SN nerves staining obtained using low or high volumes of dye solution was statistically significant (*p* = 0.0476; Fisher’s Exact test). The background colour is necessary to distinguisci between the tho nerves ot the two different legs of the same Rabbit.

	Weight (kg)	Low Volume (0.05 mL/kg)				High Volume (0.1 mL/kg)			
		SN Length (cm)	Total Dye Volume (mL)	SN Stained (cm)	SN Stained (%)	SN Length (cm)	Total Dye Volume (mL)	SN Stained (cm)	SN Stained (%)
Rabbit 1	1.6	8.8	0.08	1.5 *	17	8.7	0.16	3.0 *	34
Rabbit 2	1.6	9	0.08	1.9 *	21	9.2	0.16	2.4 *	26
Rabbit 3	1.59	9.8	0.08	2.0 *	20	9.8	0.16	4.2 *	43
Rabbit 4	1.8	9	0.09	1.9 *	21	9.3	0.18	2.9 *	31
Rabbit 5	1.55	10	0.07	1.0 *	10	9.8	0.15	1.9 *	19
Mean	1.6	8.9	0.08	1.6	17.8	9.2	0.16	2.9	31
SD	0.1	0.78	0.006	0.4	4.6	0.4	0.009	0.8	8.9

## Data Availability

The data presented in this study are available on request from the corresponding author.
