# Correction: Felisberto et al. Ultrasound-Guided Saphenous Nerve Block in Rabbits (*Oryctolagus cuniculus*): A Cadaveric Study Comparing Two Injectate Volumes. *Animals* 2022, *12*, 624

**DOI:** 10.3390/ani12121534

**Published:** 2022-06-14

**Authors:** Ricardo Felisberto, Derek Flaherty, Hamaseh Tayari

**Affiliations:** Southern Counties Veterinary Specialists (SCVS), Forest Corner Farm, Hangersley, Hampshire, Ringwood BH24 3JW, UK; ricardo.felisberto@scvetspecialists.co.uk (R.F.); derek.flaherty@scvetspecialists.co.uk (D.F.)

The authors wish to make the following correction to this paper [1]:

## Text Correction

There was an error in the original publication.

**Page 1, Abstract:** “1.62 ± 0.1 kg” needs to be replaced by “1.6 ± 0.1 kg”; the correct sentence is as follows:

Twelve hind-limbs from six cadavers (1.6 ± 0.1 kg) were included; after randomization, the SN block was performed on the right or left hind-limb, injecting 0.05 mL kg^−1^ or 0.1 mL kg^−1^ of tissue dye in lidocaine (1:50 *v*:*v*).

“19.2 ± 6.2% and 34.7 ± 16.1%” needs to be replaced by “17.8 ± 4.6% and 31.0 ± 8.9%”; the correct one is as follows:

All SNs were identified, and 17.8 ± 4.6% and 31.0 ± 8.9% of the SN length were stained using low-volume and high-volume of the dye, respectively.

**Page 6, 2.4. Statistical Analysis:** “category-1 SN stained for ≥1 but <2 cm, SN stained for ≥2 cm” needs to be replaced by “category-1 SN stained for ≥1 but ≤2 cm, category-2 SN stained for >2 cm”; the correct one is as follows:

The Fisher’s exact test was performed to determine statistical significance using two categories, category-1 SN stained for ≥1 but ≤2 cm, category-2 SN stained for >2 cm.

**Page 6, 3. Results, Lines 2–4:** “1.62 ± 0.1 kg” needs to be replaced by “1.6 ± 0.1 kg”; the correct sentence is as follows:

Due to the cadaveric preparation, it was not possible to determine the sex or age of the rabbits; however, the mean carcase weight was 1.6 ± 0.1 kg.

**Page 8, Lines 11–15:** “(0.05 mL/kg; resultant mean volume of 0.08 ± 0.007 mL)” needs to be replaced by “(0.05 mL/kg; resultant mean volume of 0.08 ± 0.006 mL)”; “(0.1 mL/kg; resultant mean volume of 0.16 ± 0.011 mL)” needs to be replaced by “(0.1 mL/kg; resultant mean volume of 0.16 ± 0.009 mL)”; the correct one is as follows:

During needling, the shaft of the needle was always visible, as was the tip when piercing the medial femoral fascia. The dye solution was injected in five hind-limbs with a low volume (0.05 mL/kg; resultant mean volume of 0.08 ± 0.006 mL) and in the other five with a high dose (0.1 mL/kg; resultant mean volume of 0.16 ± 0.009 mL).

## Error in Table

In the original publication, there was a mistake in some data for **Table 1** as published.

**Page 11, 3rd column of Table 1:** “7.8” needs to be replaced by “9.8”;

**Page 11, 4th column of Table 1:** “0.007” needs to be replaced by “0.006”;

**Page 11, 5th column of Table 1:** “1, 1.7” needs to be replaced by “1.0, 1.6”;

**Page 11, 6th column of Table 1:** “17.2, 20.7, 27.3, 20.4, 10.2, 19.2, 6.2” needs to be replaced by “17, 21, 20, 21, 10, 17.8, 4.6”;

**Page 11, 7th column of Table 1:** “8.8” needs to be replaced by “9.8”;

**Page 11, 8th column of Table 1:** “0.01” needs to be replaced by “0.009”;

**Page 11, 9th column of Table 1:** “3” needs to be replaced by “3.0”;

**Page 11, 10th column of Table 1:** “34.1, 26.7, 61.5, 32.2, 19, 34.7, 16.1” needs to be replaced by “34, 26, 43, 31, 19, 31, 8.9”; the correct Table 1 is as follows:

**Table 1:** Evaluation of the extent of nerve staining obtained by performing ultrasound-guided saphenous nerve (SN) blocks using a dye solution [permanent yellow tissue dye in lidocaine 2% (1:50)] in five rabbit cadavers (10 hind-limbs) with either low (0.05 mL/kg) or high (0.1 mL/kg) volume. Values (mean ± standard deviation) are reported for saphenous nerve total length, the length of the staining obtained, the total volume of dye solution injected for each hind-limb, and the percentage of the total length of saphenous nerves stained. SD: standard deviation; * The difference between the length of the SN nerves staining obtained using low or high volumes of dye solution was statistically significant (*p* = 0.0476; Fisher’s Exact test). The background colour is necessary to distinguisci between the tho nerves ot the two different legs of the same Rabbit.

	Weight (kg)	Low Volume (0.05 mL/kg)				High Volume (0.1 mL/kg)			
		SN Length (cm)	Total Dye Volume (mL)	SN Stained (cm)	SN Stained (%)	SN Length (cm)	Total Dye Volume (mL)	SN Stained (cm)	SN Stained (%)
Rabbit 1	1.6	8.8	0.08	1.5 *	17	8.7	0.16	3.0 *	34
Rabbit 2	1.6	9	0.08	1.9 *	21	9.2	0.16	2.4 *	26
Rabbit 3	1.59	9.8	0.08	2.0 *	20	9.8	0.16	4.2 *	43
Rabbit 4	1.8	9	0.09	1.9 *	21	9.3	0.18	2.9 *	31
Rabbit 5	1.55	10	0.07	1.0 *	10	9.8	0.15	1.9 *	19
Mean	1.6	8.9	0.08	1.6	17.8	9.2	0.16	2.9	31
SD	0.1	0.78	0.006	0.4	4.6	0.4	0.009	0.8	8.9

## Figure 6 Legend

**Page 9, Lines 13–15:** “(8.8 cm)” needs to be replaced by “(9 cm)”; “(2.4 cm)” needs to be replaced by “(1.9 cm)”; the correct one is as follows:

(**d**) The SN was completely removed from the cadaver (from where it branched off the FN to the proximal stifle, where it deepened to innervate the articular capsule) for the evaluation of its total length (9 cm) and extent of staining from the tissue dye solution (1.9 cm).

## Figure 7 Legend

**Page 10, Lines 13–16:** “(7.8 cm)” needs to be replaced by “(8.7 cm)”; “(4.8 cm)” needs to be replaced by “(4.2 cm)”; the correct one is as follows:

(**d**) The SN was completely removed from the cadaver (from where it branched off the FN to the proximal stifle, where it deepened to innervate the articular capsule) for the evaluation of its total length (8.7 cm) and extent of staining from the tissue dye solution (4.2 cm).

The authors apologize for any inconvenience caused and state that the scientific conclusions are unaffected. The original publication has also been updated.
